# Interaction of RECQ4 and MCM10 is important for efficient DNA replication origin firing in human cells

**DOI:** 10.18632/oncotarget.6342

**Published:** 2015-11-17

**Authors:** Maciej Kliszczak, Hana Sedlackova, Ganesha P. Pitchai, Werner W. Streicher, Lumir Krejci, Ian D. Hickson

**Affiliations:** ^1^ Center for Chromosome Stability and Center for Healthy Aging, Department of Cellular and Molecular Medicine, University of Copenhagen, Copenhagen N, Denmark; ^2^ National Centre for Biomolecular Research and Department of Biology, Masaryk University, and St Anne's University Hospital, Brno, Czech Republic; ^3^ Novo Nordisk Foundation Centre for Protein Research, Faculty of Health and Medical Sciences, University of Copenhagen, Copenhagen N, Denmark; ^4^ Present address: Novozymes A/S, 2880 Bagsvaerd, Denmark

**Keywords:** DNA replication, RecQ helicases, minichromosome maintenance proteins, Chromosome Section

## Abstract

DNA replication is a highly coordinated process that is initiated at multiple replication origins in eukaryotes. These origins are bound by the origin recognition complex (ORC), which subsequently recruits the Mcm2-7 replicative helicase in a Cdt1/Cdc6-dependent manner. In budding yeast, two essential replication factors, Sld2 and Mcm10, are then important for the activation of replication origins. In humans, the putative Sld2 homolog, RECQ4, interacts with MCM10. Here, we have identified two mutants of human RECQ4 that are deficient in binding to MCM10. We show that these RECQ4 variants are able to complement the lethality of an avian cell *RECQ4* deletion mutant, indicating that the essential function of RECQ4 in vertebrates is unlikely to require binding to MCM10. Nevertheless, we show that the RECQ4-MCM10 interaction is important for efficient replication origin firing.

## INTRODUCTION

The control of the initiation of DNA replication is important for the timely and faithful duplication of the genome. DNA replication initiation occurs in two defined steps, which are conserved amongst vertebrate species. In the first step, conducted prior to S-phase, the origin recognition complex (ORC) binds to specific replication origins. With the exception of certain lower eukaryotes, these origins do not have a well-defined DNA sequence [1]. It is possible that other structural features of the DNA define an origin; for example, specific chromatin marks, or the presence of DNA secondary structures in the proximity of the replication origin. Indeed, the presence of G-quadruplexes has recently been proposed to play an important role in the initiation of DNA replication in chicken cells [2, 3]. The origin-bound ORC directs the recruitment of Cdc6 and Cdt1, which subsequently allows loading of the heterohexameric replicative helicase comprising the Mcm2-7 proteins [4, 5]. In the second step, origin activation depends on the Cdc7-Dbf4 (DDK) and S-phase cyclin dependent kinases (S-CDK) (reviewed in [6]). In budding yeast, DDK phosphorylates the Mcm2-7 helicase in Sld3-dependent manner (TRESLIN in humans) leading to its activation through recruitment of Cdc45 [7]. Following this, S-CDK phosphorylates Sld2 (RECQ4 in humans) and Sld3, facilitating the binding of the BRCT domain-containing protein, Dpb11 (TOPBP1 in humans) and recruitment of the GINS complex to the already assembled origin-protein complex [8, 9]. Similarly, phosphorylation of TRESLIN is important for interaction with TOPBP1 and for normal S-phase progression in human and *Xenopus* systems [10, 11]. Activation of the replicative helicase occurs through the formation of the CMG complex comprising the Cdc45-Mcm2-7-GINS, which possesses robust DNA unwinding activity [12, 13]. This pre-initiation complex then recruits Mcm10 which serves as a chaparone for Polymerases α and δ leading to the formation of active replication forks and subsequently to genome duplication (reviewed in [14]).

RECQ4, together with RECQ1, BLM, WRN and RECQ5, belongs to the RecQ helicase family required for multiple aspects of DNA metabolism. The N-terminal region of RECQ4, not associated with the conventional RecQ helicase core domain, is the only region to share homology with Sld2 [15-17]. Mutations of human *RECQ4* have been linked to three distinct clinical syndromes: Rothmund-Thomson type II, RAPADILINO and Baller-Gerold. These disorders share some overlapping phenotypes, including radial ray defects, facial dysmorphia and a predisposition to the development of cancer, especially osteosarcoma. In addition, RAPADILINO and Baller-Gerold individuals show skeletal defects, such as a malformed patella and craniosynostosis, respectively (reviewed in [18]). Interestingly, most of the mutations found in affected individuals occur outside of the Sld2-like N-terminal domain of the protein, but instead usually affect the helicase and C-terminal domain containing the RQC motif of RECQ4. This suggests that a lack of DNA unwinding mediated by RECQ4 might be important in the etiology of these diseases ([19, 20] and reviewed in ([18]).

RECQ4 and its homologs appear to play roles in both DNA repair and DNA replication [15, 16, 20-36]. The N-terminal Sld2-like domain is apparently the only region of RECQ4 that is required for DNA replication in vertebrates [15, 16, 31, 35, 36]. This domain contains a disordered region required for interaction with DNA substrates that possibly form at origins of replication, including G-quadruplexes and Y-DNA [37, 38]. Moreover, the first 54 amino acids of RECQ4 fold into a homeodomain-like DNA binding site [39]. RECQ4 binds to origins of DNA replication at the G1/S-phase boundary, and its depletion leads to significant reduction in the frequency of origin firing in human cells [35]. RECQ4 depletion does not affect replication elongation, in line with what is known about Sld2 in budding yeast [35, 40]. Human RECQ4 interacts physically with MCM10 and CTF4, and is required for the association of these proteins with replication origins. In turn, MCM10 is required for RECQ4 localisation to origins [41]. Moreover MCM10 bridges RECQ4 association with the MCM2-7 helicase, suggesting that the MCM10-RECQ4 complex might be critical for origin activation [32].

Yeast Mcm10 was first identified in genetic screens for mutants defective in DNA replication and mini-chromosome maintenance [42, 43]. Mcm10 is an evolutionary conserved factor with a centrally located DNA binding zinc finger motif that is necessary for cell viability in budding yeast [44]. In vertebrates, MCM10 physically interacts with the MCM2-7 helicase and TOPBP1, and is required for the loading of POLα, POLδ and RPA to replication origins, as well as for stabilization of POLα at active replication forks [25, 45-48]. Budding yeast Mcm10 is recruited to the chromatin after formation of the pre-initiation complex in a CDK-dependent step and this recruitment requires prior loading of Cdc45, Sld2, Sld3, GINS and Dpb11, suggesting that Mcm10 functions in the latter stages of origin activation [47, 49].

In order to define the role of the interaction between RECQ4 and MCM10, we identified the MCM10 interaction interface on RECQ4. We generated two RECQ4 mutants deficient in MCM10 binding, and have analyzed the consequences of the loss of the RECQ4-MCM10 interaction on cell proliferation, cell cycle progression and replication origin firing. We found that RECQ4 variants lacking the MCM10 interaction domain were able to associate with replication factors such as the MCM7 and CDC45, but one of them showed a much reduced affinity for binding TOPBP1. Surprisingly, both mutants were able to largely support the viability of *RECQ4*-deficient DT40 cells, indicating that the interaction of RECQ4 with MCM10 is not essential for cell viability. Nevertheless, we found that this interaction is required for robust origin firing in vertebrate cells.

## RESULTS

### RECQ4 and MCM10 interact directly *in vitro* and in human cells

**Figure 1:**
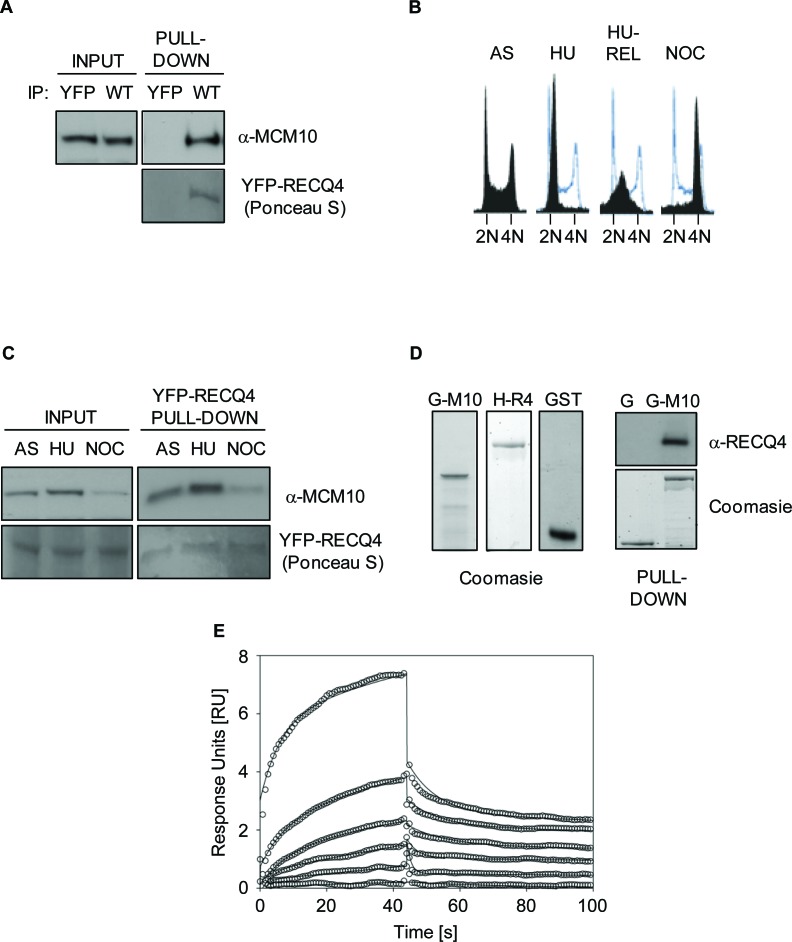
RECQ4 interacts directly with MCM10 **A.** Immunoblot analysis for MCM10 in anti-YFP immunoprecipitates from U20S cells expressing either YFP alone or YFP-RECQ4 (WT). Ponceau S staining was used to confirm the presence of YFP-RECQ4 in the precipitates. **B.** Flow cytometry analysis for asynchronous (AS), hydroxyurea-arrested (HU), HU-arrested cells released into S-phase (HU-REL) and nocodozole arrested U20S cells (NOC). DNA was stained using propidium iodide. The solid black traces show the distribution of cells, and the blue traces show the outline of the control (AS) sample for comparison. **C.** Immunoblot analysis for MCM10 in anti-YFP-RECQ4 immunoprecipitates from the cells shown in panel B. Loading is shown using Ponceau S staining. **D.** Coomassie blue stained polyacrylamide gels showing purified GST-MCM10 (G-M10), His-RECQ4 (H-R4) or GST alone (G and GST). The panel on the right shows immunoblot analysis for RECQ4 in GST pull-down samples, together with a Coomassie stained gel showing the loading control for the GST proteins. **E.** Binding kinetics analysis of RECQ4 interacting with immobilized MCM10 using surface plasmon resonance (Biacore). Open circles represent the experimental data at different RECQ4 concentrations (1, 0.5, 0.25, 0.125, 0.06, and 0.03 μM) and the solid lines represent the fit of the data to a two state model. The dissociation constant (*K*_d_) was determined by the ratio of *k*_on_ and *k*_off_ and was found to be 170±200 nM.

Previously, Xu and colleagues reported that a physical interaction exists between MCM10 and RECQ4 that requires the first 100 amino acids of RECQ4 [32]. In order to explore the cellular roles of the RECQ4-MCM10 interaction, we set out to map the MCM10 binding interface on RECQ4 molecule in finer detail. First, we confirmed that a YFP-RECQ4 fusion protein could interact with MCM10 in living cells. For this, we transiently expressed YFP-RECQ4 (or YFP alone as a control) in U2OS cells, and then immunoprecipitated the YFP proteins using GFP-Trap beads. We efficiently precipitated MCM10 together with RECQ4 from cells expressing YFP-RECQ4, but not those expressing YFP alone (Figure 1A). We then addressed at which stage of the cell cycle these two proteins interact. For this, we precipitated YFP-RECQ4 from asynchronous (AS), hydroxyurea (HU) arrested, and nocodazole (NOC) arrested cells. We observed that the RECQ4-MCM10 complex formed at all stages of the cell cycle and its abundance broadly mirrored that of the level of MCM10 protein (Figure 1B, 1C). Next, we analyzed whether RECQ4 and MCM10 are able to interact directly using purified recombinant proteins. We observed that GST-MCM10 could precipitate HIS-RECQ4, although GST alone could not, suggesting a direct physical interaction between RECQ4 and MCM10 (Figure 1D). To confirm this finding and to obtain an estimate of the *K*_d_ of the RECQ4-MCM10 interaction, we utilized Surface Plasmon Resonance. For this, the interaction of RECQ4 with GST-MCM10 immobilized on a CM3 chip was analyzed. This analysis confirmed that RECQ4 and MCM10 directly interact with a *K*_d_ value of approximately 170 nM (Figure 1E).

### Identification of the MCM10 interaction interface on RECQ4

**Figure 2:**
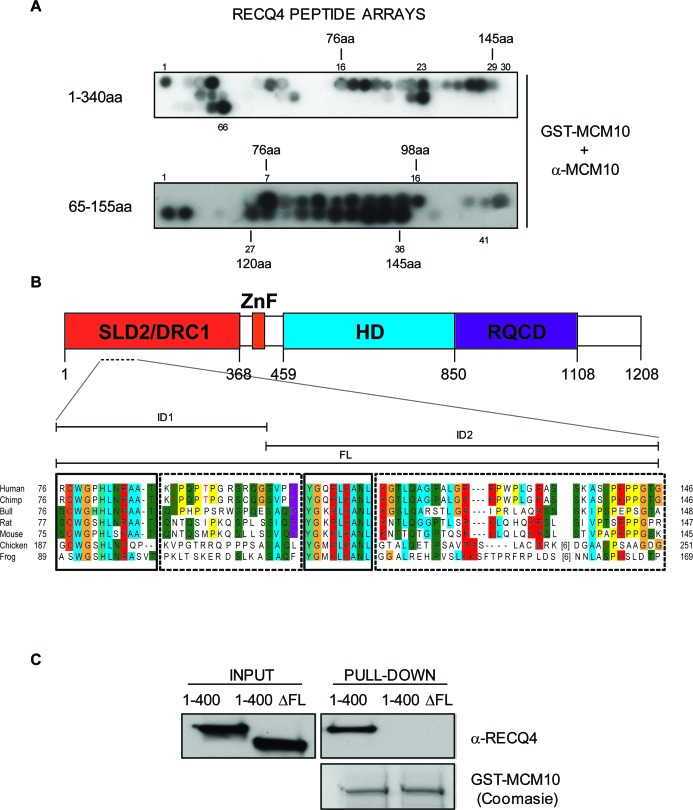
Mapping of the RECQ4-MCM10 interaction domain in RECQ4 **A.** Immunoblot analysis of two RECQ4 peptide arrays (residues 1-340; upper panel and 65-155; lower panel) after incubation of the membrane with purified GST-MCM10 and anti-MCM10 antibodies. **B.** Sequence alignment of RECQ4 proteins from various species in the region of RECQ4 where MCM10 binds. Identical or very similar residue groups are colored. The solid boxes show highly conserved domains and the dashed boxes less highly conserved domains. The positions of the ID1, ID2 and FL regions are indicated above. The putative SP/TP target sites for Cdk's are shown in red text. **C.** Immunoblot analysis using anti-RECQ4 antibodies of protein pull-down using purified GST-MCM10. The input contained either MBP-RECQ4 1-400-HIS fragment or MBP-RECQ4 1-400-HIS with the ΔFL deletion, as indicated.

To accurately map the MCM10 binding interface on RECQ4, we utilized peptide arrays. The first 100 amino acids of RECQ4 have been shown to be necessary for the interaction between the two proteins; however, it was not clear whether these residues were also sufficient for the interaction. Hence, we tested membrane-bound peptide arrays spanning amino acids 1-340 of RECQ4 (10 amino acids per peptide; a 5 amino acid overlap between each peptide). We then incubated the membrane with purified GST-MCM10 and analyzed any signal detected using an anti-MCM10 antibodies. The largest putative region of interaction spanned peptides 16-29, which corresponds to amino acids 76-145 of RECQ4 (Figure 2A, 2B, top panel, region designated FL). This region appeared to comprise two sub-domains important for MCM10 binding between peptides 16-23 (designated ID1) and 25-29 (designated ID2). These regions correspond to amino acid positions 76-120 and 125-145, respectively. To further refine this analysis, we utilized a second peptide array spanning amino acids 66-155 (10 amino acids per peptide with a 9 amino acid overlap). This analysis provided further evidence for two distinct interfaces (amino acids 76-97 and 120-145) able to bind MCM10 (Figure 2A, 2B). Furthermore, bioinformatic analysis of the ID1 and ID2 interaction regions showed that they are well conserved in RECQ4 proteins across different species (Figure 2B).

### Disruption of the RECQ4-MCM10 interaction

**Figure 3:**
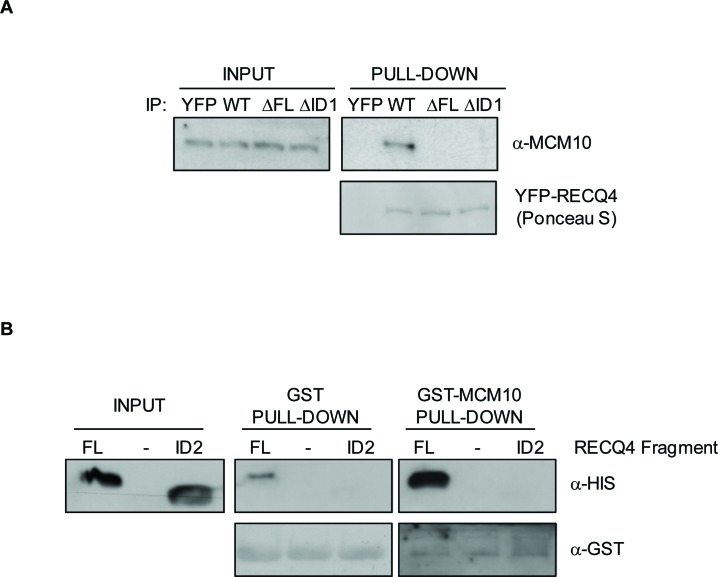
Characterisation of the RECQ4-MCM10 interaction in human cells **A.** Immunoblot analysis using anti-MCM10 antibodies of protein immunoprecipitated using GFP-trap beads from U20S cells expressing YFP alone, YFP-RECQ4, YFP-RECQ4-ΔFL or YFP-RECQ4-ΔID1. The precipitated YFP proteins were stained with Ponceau S, as indicated. **B.** Immunoblot analysis of His-tagged RECQ4 fragments (FL or ID2) pulled-down *in vitro* using either GST alone or GST-MCM10. Anti-GST immunoblot was used as a loading control.

Next, we addressed whether deletion of the putative binding interface on RECQ4 could disrupt the interaction with MCM10. We generated a truncated variant of RECQ4 (RECQ4-ΔFL; with deletion of amino acids 75-145 in a fragment comprising residues 1-400 fused to MBP) (Figure 2B, 2C). Deletion of residues 75-145 in RECQ4 resulted in loss of the binding to MCM10, confirming that this region of RECQ4 contains residues necessary for the interaction (Figure 2C). We then asked if either or both of the aforementioned interfaces (ID1 and ID2) were important for the RECQ4-MCM10 interaction. For this, we generated two truncated YFP fusion protein variants of RECQ4; either RECQ4 lacking the FL domain (RECQ4-ΔFL) or RECQ4 lacking just the ID1 interface (RECQ4-ΔID1). Following transient expression in U2OS cells, the YFP fusion proteins were immunoprecipitated and associated proteins identified by immunoblotting. We observed that deletion of either the FL or the ID1 domain greatly diminished binding of RECQ4 to MCM10, as compared to wild-type RECQ4 (Figure 3A). This analysis indicated that ID1 was necessary for the interaction of RECQ4 with MCM10 and that ID2 alone cannot suffice. Indeed, by analysis of recombinant FL and ID2 fragments prepared from *E.coli*, we observed that the FL domain, but not the ID2 domain, could interact with MCM10. These data indicate that the ID2 motif plays a minor role, if any, in directing the binding of RECQ4 to MCM10 (Figure 3B).

Interestingly, within the region of RECQ4 important for MCM10 binding there are three putative cyclin-dependent kinase (CDK) recognition sites at S89, T93 and T139. Previously, these sites have been shown to weakly affect the formation of the RECQ4-MCM10 complex *in vitro* when studies were conducted under specific (high salt) conditions [32]. Although these sites are not conserved between human RECQ4 and homologues in other species, we tested whether S89, T93 and T139 might be important for the formation of the RECQ4-MCM10 complex in human cells. For this, we expressed versions of RECQ4 containing single or multiple alanine (non-phosphorylatable) or glutamic acid (to mimic phosphorylation) substitutions at these sites in U2OS cells. However, we observed no effect on the interaction with MCM10 (data not shown), suggesting that if modification of RECQ4 at these particular sites occurs in living cells, it plays a very minor role in the regulation of the RECQ4-MCM10 interaction.

### Role of the RECQ4-MCM10 interaction in cells

**Figure 4:**
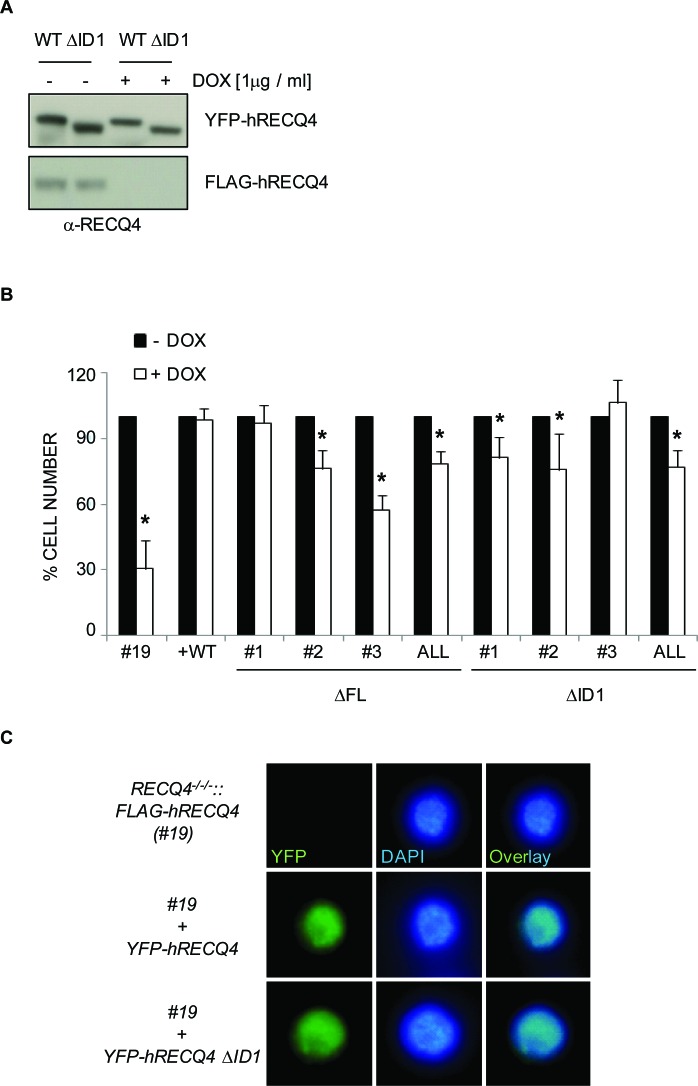
Analysis of RECQ4 variants in chicken DT40 cells (clone #19) containing a conditional RECQ4 mutation **A.** Western blotting analysis using anti-RECQ4 antibodies of DT40 extracts from cells grown with (+) or without (−) doxycycline. Note that expression of the transgene (FLAG-RECQ4) is repressed by doxycycline but the transfected YFP-RECQ4 is not. **B.** Quantification of cell number 72 hours after addition of doxycycline (+) or mock treated (−). Three independent clones for each of the mutant RECQ4 variants (ΔFL or ΔID1) are shown, together with the average of the three (ALL). Error bars represent SD from at least three independent experiments. **C.** Direct fluorescence analysis of cells expressing no human RECQ4 (top panel), YFP-RECQ4 (middle) or YFP-RECQ4-ΔID1 (bottom). Note the localization of the YFP proteins to the nucleus, as shown by DAPI staining of DNA.

*RECQ4* is an essential gene in mice, frogs, fruit flies and chicken cells, and is proposed to play a key role in the initiation of DNA replication [15, 31, 33, 35, 50, 51]. We hypothesised that the RECQ4-MCM10 interaction might be essential for the establishment of functional replicons through facilitating robust origin activation, a role proposed for the yeast homologues of these proteins; Sld2/Drc1 and Mcm10/Cdc23 (reviewed in [52]). To study whether the interaction between human RECQ4 and MCM10 is essential for cell viability, we took advantage of a conditional *RECQ4* gene deletion in the chicken DT40 cell line [31]. *RECQ4^−/−/−^::hFLAG-RECQ4* cells do not express endogenous chicken *RECQ4*, but maintain viability due to the presence of a human *FLAG-RECQ4* transgene under the control of a doxycycline-regulated promoter (Tet-OFF system). It has been shown previously that within 48 hours of repression of human FLAG-RECQ4 protein expression, the RECQ4-deficient chicken cells cease to proliferate [31]. We generated stable DT40 cell clones expressing either YFP-RECQ4-ΔFL or YFP-RECQ4-ΔID1 (or YFP-RECQ4 wild-type as a control) from the CMV promoter in the *RECQ4^−/−/−^::hFLAG-RECQ4* background. As expected, we could efficiently downregulate expression of the *FLAG-RECQ4* transgene, but not the transfected human *YFP-RECQ4*, using doxycycline (Figure 4A and data not shown). To address whether these variants could support chicken cell viability in the absence of any wild-type RECQ4, the expression of *FLAG-hRECQ4* was repressed by addition of doxycycline, and cell number was monitored over the next 72 hours. As expected, the DT40 cells lacking the *YFP-RECQ4* transgene ceased to proliferate, unlike the cells expressing YFP-RECQ4 (Figure 4B). Interestingly, we could detect a partial, but significant, proliferation deficit in cells expressing RECQ4-ΔFL or RECQ4-ΔID1 (Figure 4B). We conclude that the MCM10 interaction domain in RECQ4 is not essential for cell viability, but is required to support a robust level of cell proliferation. One possible explanation for this is that the YFP-RECQ4-ΔFL and -ΔID1 proteins might display an altered pattern of nuclear localisation; however, we observed that this was indistinguishable from that of wild-type YFP-RECQ4 (Figure 4C and data not shown).

**Figure 5:**
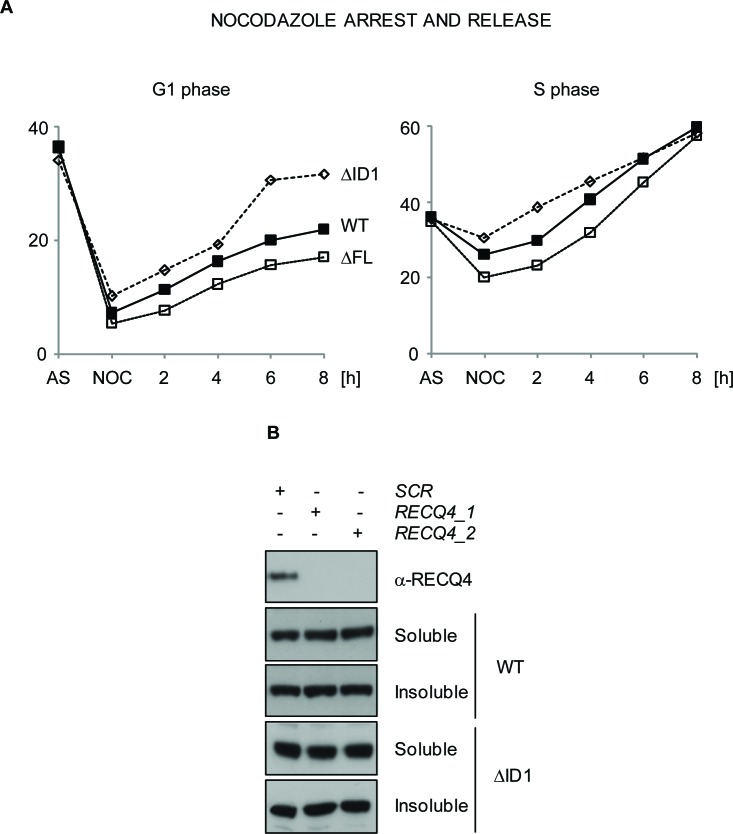
RECQ4-MCM10 interaction is required for normal cell cycle progression but not for RECQ4 chromatin localization **A.** Analysis of cell cycle transit time using flow cytometry. DT40 cells expressing YFP-RECQ, YFP-RECQ4-ΔFL or -ΔID1, as indicated, were arrested with nocadazole (NOC) and released into the next cell cycle for up to 8 hours. Points are the mean of three independent experiments. **B.** Western blotting analysis of cell extracts from U20S cells transfected with either of two independent siRNAs targeting RECQ4 (1 and 2) or a scrambled siRNA control (SCR). Note that the endogenous RECQ4 is depleted (top panel) but the transfected, siRNA-resistant WT RECQ4 WT and RECQ4-ΔID1, are not.

To examine the possibility that the RECQ4-ΔMCM10 expressing cells might have altered cell cycle distribution, we repressed the *FLAG-hRECQ4* transgene in cells expressing the YFP-RECQ4-ΔFL variant, and then arrested the cells in prometaphase using nocodazole. We then monitored cell cycle progression using flow cytometry following release of cells from this arrest into the next cell cycle (Figure 5A). Cells expressing the ΔFL mutant were proficient for an arrest in prometaphase induced by nocodazole, but following release showed a small but consistent alteration in the rate of progression through the G1 and S phases (Figure 5A and data not shown). This was consistent with the observed proliferation defect. From this we conclude that the interaction between RECQ4 and MCM10 influences normal cell cycle progression.

### The RECQ4-MCM10 interaction is required for robust replication origin firing

To explain the observed phenotypes we explored several possibilities. We hypothesised that loss of the interaction with MCM10 might affect the chromatin localisation of RECQ4, its ability to form stable complexes with other replication factors, or the efficiency of replication origin firing. Previous studies in human cells revealed that RNAi-mediated depletion of MCM10 in HeLa cells disrupts the localisation of RECQ4 to chromatin [53]. Therefore, we addressed whether a RECQ4 variant unable to bind MCM10 could be still recruited to the chromatin in living cells. We transiently expressed wild-type and RECQ4 variant lacking the MCM10 interaction domain in U2OS cells that had been exposed to siRNAs that downregulate the endogenous *RECQ4*. We then performed a sub-cellular fractionation procedure. We could detect robust chromatin association for the RECQ4-ΔID1 (Figure 5B). This indicated that RECQ4 binding to the chromatin was largely independent of the MCM10 interaction domain and suggested, in contrast to a previous report, that RECQ4 does not require MCM10 for its chromatin localisation [53].

**Figure 6:**
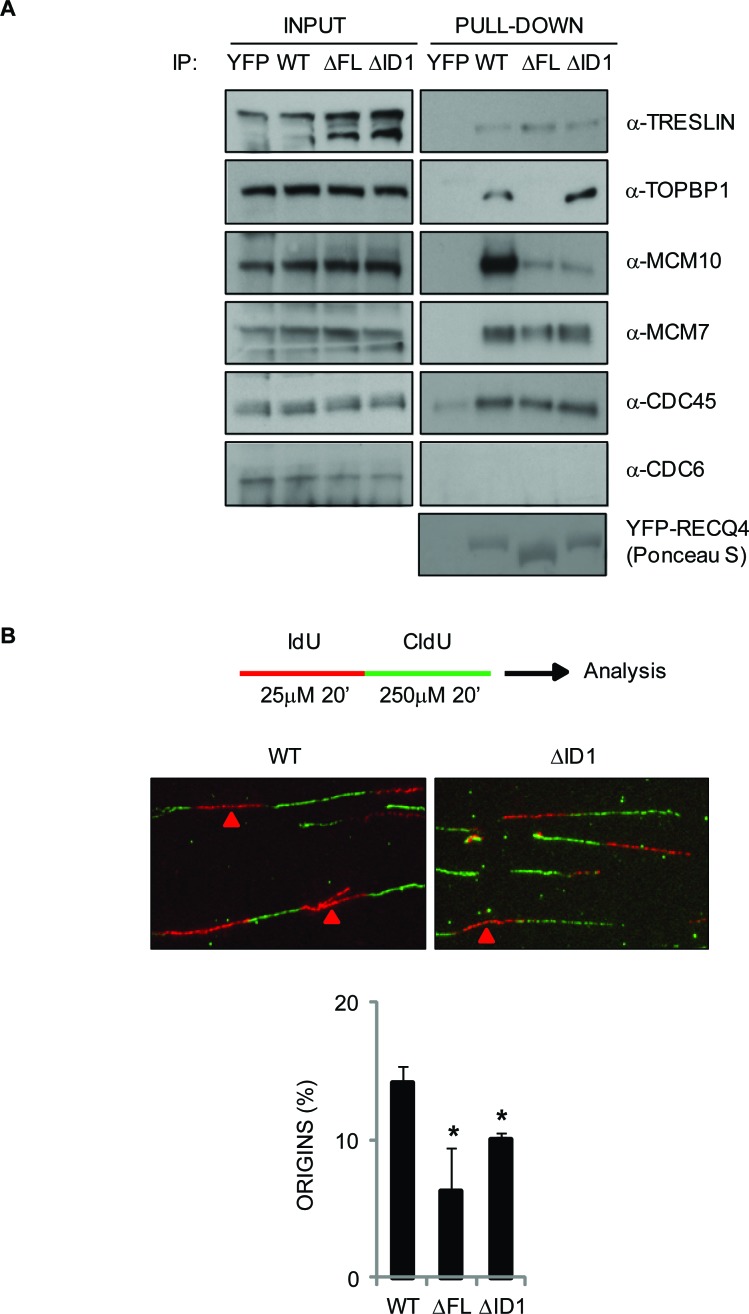
Analysis of protein complexes associated with the truncated RECQ4 variants and their ability to support normal DNA replication in DT40 cells **A.** Western blotting analysis of proteins pulled-down using YFP alone, YFP-RECQ4, or the ΔFL and ΔID1 variants. The input samples for each pull down are shown. The proteins analyzed are shown on the right. Ponceau S staining was used to confirm a similar loading of the YFP-RECQ4 proteins. **B.** DNA replication analysis on isolated DNA fibres. The upper cartoon shows the sequential labeling protocol using IdU (red) and CldU (green) nucleoside analogues. The middle panel shows representative fibres. Red arrows devote red tracts flanked by green tracts, denoting the positions of replication origins. The lower panel shows relative frequency of origin firing events. Error bars represent SD from three independent experiments. *=*p* < 0.05.

MCM10 is known to be important for the association of RECQ4 with the MCM2-7 helicase [32], for the assembly of CMG complexes (CDC45-MCM2-7-GINS), and for robust origin firing in human cells [35, 53]. Therefore, we analyzed whether the loss of the MCM10 binding domain affected the ability of RECQ4 to form specific protein complexes. We expressed YFP alone, YFP-RECQ4 wild-type, YFP-RECQ4-ΔFL and YFP-RECQ4-ΔID1 in U2OS cells, immunoprecipitated the YFP-containing proteins, and then immunoblotted for known replication factors. We observed that, as expected, the two mutant RECQ4 variants did not co-immunoprecipitate MCM10 (Figure 6A). Nevertheless, these RECQ4 variants were able to co-immunoprecipitate several other DNA replication factors, including MCM7, TRESLIN and CDC45 (Figure 6A). These data are inconsistent with a previous report indicating that MCM10 is required for the association of RECQ4 with the MCM2-7 helicase, but consistent with the fact that budding yeast Sld2 can interact directly with the replicative helicase [32, 54]. Interestingly, the ΔID1 protein, but not the ΔFL variant, could efficiently co-precipitate TOPBP1 (Figure 6A). We conclude that the ΔFL variant shows altered interaction with MCM10 and TOPBP1, but retains that ability to bind several other DNA replication factors.

Next, we analyzed whether the RECQ4-MCM10 interaction might influence the efficiency of replication origin firing. For this we utilized a dual labelling protocol to define the position of active replicons in isolated DNA fibres. We pulse labelled the DNA of cells expressing the wild-type or mutant RECQ4 proteins with IdU and CldU, extracted DNA fibres and quantified the frequency of different replication structures (Figure 6B). We could readily observe labelling patterns indicative of replication origins, ongoing forks and termination events. We observed a significantly reduced number of origin-containing labelled tracts in cells expressing the mutant variants compared to wild-type RECQ4 (Figure 6B). These data are consistent with the proposed role of RECQ4 in the initiation of DNA replication and indicate that the interaction between MCM10 and RECQ4 is important for supporting the efficient firing of replication origins.

## DISCUSSION

Our analysis of RECQ4-MCM10 interaction has identified two regions within the Sld2-like N-terminus of RECQ4 that are essential for MCM10 binding. We showed that the interaction between RECQ4 and MCM10 is partially required for viability of chicken cells lacking endogenous RECQ4, and for normal replication origin firing. However, this interaction is not required for localisation of RECQ4 with chromatin or its association with replication factors such as MCM7, TRESLIN or CDC45. These data indicate that the formation of RECQ4-MCM10 complex plays important roles in the progression of the cell cycle and normal cell proliferation through activation of active origins of replication during S phase. Moreover, the FL domain was shown to contain a sequence important for efficient formation of a RECQ4-TOPBP1 complex.

Recent work suggested that yeast Sld2 binds to origins of replication before Mcm10, and that this prior binding is a prerequisite for the association of MCM10 with chromatin [47]. Budding yeast Mcm10 has been proposed to be involved in a novel step in replication initiation after the assembly of CMG complexes onto origins, suggesting a role in the latter stages of the initiation of DNA replication [46, 55]. In contrast, in HeLa cells, depletion of MCM10 causes loss of RECQ4 chromatin localisation [53], suggesting a different mechanism may be operating in human cells. However, we observed that human RECQ4 could be recruited to the chromatin independently of MCM10, indicating that loading of RECQ4 onto chromatin may occur prior to recruitment of MCM10. The discrepancies between our study and results of Xu and colleagues [32] could be explained by different approaches used. We analyzed an interaction-defective mutant that allowed us to explore directly the relationship between MCM10 and RECQ4, whereas Xu and colleagues used MCM10 depletion. It is plausible that in MCM10-depleted cells, the structure of replication origins or the composition of the origin-bound protein complex might be fundamentally different from those in the control cells, thus preventing the recruitment of RECQ4.

Recent work by the Pospiech and Krejci laboratories, has described multiple DNA binding interfaces in RECQ4 [37-39]. Interestingly, these DNA binding regions lie within the Sld2-like domain of RECQ4 and show strong preferential binding towards G4 quadruplexes. Such sequences are potetially also found in the proximity of origins of DNA replication [3, 37]. Although our unpublished data indicate that the FL interaction domain severely affects the DNA binding affinity of RECQ4 *in vitro,* it is unlikely that this mutant behaves similarly in the cellular context. It would be surprising if significant loss of the DNA binding activity would only partially affect the cellular roles of this essential helicase, as we observed in our experiments. An obvious explanation for this finding is that there is likely to be at least one additional binding domain in the RECQ4 molecule.

Patients bearing *RECQ4* mutations mainly express RECQ4 C-terminal truncations sparing the N-terminal Sld2-like domain that is essential for cell viability (reviewed in [18]). This strongly suggests that the essential functions of RECQ4 are associated with initiation of DNA replication and are not dependent on its helicase activity [15, 31, 32, 35]. Indeed, *RECQ4*-deficient adult mice show hematopoietic abnormalities, including bone marrow failure caused by S phase arrest and apoptosis, which can be rescued by expression of a helicase-dead mutant of RECQ4 [56]. We hypothesised that the loss of the RECQ4-MCM10 interaction site that is located within the essential domain of RECQ4 might result in cell lethality. We found that the RECQ4-MCM10 interaction was required for normal cell cycle progression and for robust origin firing; however, it was not essential for viability, suggesting that if the essential functions of RECQ4 helicase are associated with initiation of DNA replication, they are not mediated *via* an interaction with MCM10. Interestingly, previous observations using the chicken cell system indicated that the entire 1-496 amino acid N-terminal domain is required for cell viability. Hence, the RECQ4-ΔID1 and RECQ4-ΔFL mutants we studied represent the only variants described thus far lacking a region of the N-terminal domain that are able to rescue the viability of vertebrate cells lacking endogenous RECQ4.

The mild phenotype of cells expressing RECQ4 variants that cannot bind MCM10 is likely explained by the observation that these variants can still interact with other replication factors, including MCM7, TRESLIN and CDC45. The ability of the ΔFL mutant to rescue viability of DT40 cells, while lacking an interaction with TOPBP1, suggests that RECQ4 might act in a mechanistically distinct way than the Sld2/Drc1, as their interactions with Dpb11/Rad4 is essential for viability in yeast [8, 9]. However, at this stage and without further dissection of the essential cellular functions of RECQ4, this remains conjecture. One final outcome of our study is based on the finding that the ΔFL, but not the ID1, mutant failed to efficiently form a complex with TOPBP1, but was still able to rescue viability of DT40 cells. This suggests that the ID2 domain likely contains a TOPBP1 interacting motif. Future studies on this putative domain would likely be valuable for our understanding the functions of RECQ4 in human cells.

## MATERIALS AND METHODS

### Gene cloning

The human GST-MCM10 construct was a kind gift from Dr. Yilun Liu (pGEX-4T-1-MCM10) [32]. Human *RECQ4* cDNA was cloned into pEYFPC1 (Clontech) between *SalI* and *XbaI* sites. Fragments of human RECQ4 corresponding to amino acids 76-145 (RECQ4-MCM10 FL) and amino acids 105-145 (RECQ4-MCM10 ID2 domain) were cloned into pET14b (Novagen) *via* pGEMT-Easy (Promega) with 5′-GGA TCC GCG CTG CTG GGG GCC C-3′, 5′-GGA TGT ACC TGG GGG CTT TGG GG-3′ and 5′-GGA TCC GGG GCA GCG GCT CAA-3′ and 5′-GGA TCC TGT ACC TGG GCG CTT TGG GG-3′ oligunucleotides, respectively, using Takara LA Taq polymerase (Clontech). To create deletion of MCM10 interaction domain inhuman RECQ4-ΔFL and RECQ4-ΔID1 extra *SacII* restriction site was introduced by site directed mutagenesis in the pEYFPC1-RECQ4 vector with 5′-GGTACAGGGCCTGTCCCCTCCGCGGCAGAAA AAGTCAGTGATGAGCCTCC-3′,5′-GGAGGCTCATCACTGACTTTTTCTGCCGCGGAGGGGACAGGCCCTGTACC-3′ and 5′-GGC GGA GCC GCC AGG CCG CGG TGC CGG ACT ACG-3, 5′-CGT AGT CCG GCA CCG CGG CCT GGC GGC TCC GCC-3′oligonucleotides, respectively (mutated bases are underlined). Mutated plasmids were then recovered from *E.coli* TOP10 (Life Technologies), digested with *SacII* and re-ligated to create appropriate deletion. To generate alanine and glutamic acid substitutions of S89, T93 and T139 the point mutations were introduced by site directed mutagenesis in the pEYFPC1-RECQ4 vector with the following oligonucleotides: S89A 5′-CGG GCT GCG ACC AAG GCA CCA CAG CCT ACG CCA G-3′ and 5′-CTG GCG TAG GCT GTG GTG CCT TGG TCG CAG CCC G; S89E 5′-GAA TCG GGC TGC GAC CAA GGA GCC ACA GCC TAC GCC AGG G-3′ and 5′-CCC TGG CGT AGG CTG TGG CTC CTT GGT CGC AGC CCG ATT C-3′; T93A 5′-GAC CAA GAG TCC ACA GCC TGC TCC AGG GCG GAG CCG CCA GGG-3′ and 5′-CCC TGG CGG CTC CGC CCT GGA GCA GGC TGT GGA CTC TTG GTC-3′; T139A 5′-GCC TCA TCT AAG GCA TCC GCC CCA AAG CCC CCA G-3′ and 5′-CTG GGG GCT TTG GGG CGG ATG CCT TAG ATG AGG C-3′; T139E 5′-GCC TCA TCT AAG GCA TCC GAG CCA AAG CCC CCA GGT AC-3′ and 5′-GTA CCT GGG GGC TTT GGC TCG GAT GCC TTA GAT GAG GC-3′; S89A/T93E 5′-GAC CAA GGC ACC ACA GCC TGC TCC AGG GCG GAG CCG CCA GGG-3′ and 5′-CCC TGG CGG CTC CGC CCT GGA GCA GGC TGT GGT GCC TTG GTC-3′ using the pEYFPC1-RECQ4 S89A as a template; S89E/T93E 5′-GCG ACC AAG GAG CCA CAG CCT GAG CCA GGG CGG AGC CG-3′ and 5′-CGG CTC CGC CCT GGC TCA GGC TGT GGC TCC TTG GTC GC-3′ using pEYFPC1-RECQ4 S89E as a template (mutated bases are underlined). Site directed mutagenesis reactions were performed using Phusion High-Fidelity Polymerase (Thermo Scientific). The MBP-RECQ4-HIS 1-400 and 1-400 ΔFL was generated by sub-cloning of RECQ4 cDNA fragment from pEYFPC1-RECQ4 WT or ΔFL constructs into pMAL-c2x-9HIS.

### Antibodies

Primary antibodies used were as follows: rat anti-BrdU (clone BU1/75 ICR1 for CldU detection, Abcam) at 1:40, mouse anti-BrdU (clone B44 for IdU detection, Beckton Dickinson) at 1:2, mouse anti-human CDC6 (sc-9964, Santa Cruz) at 1:1000, rabbit anti-human CDC45 (15678-1-AP, Protein Tech Group) at 1:500, mouse anti-HIS (clone HIS-1, Sigma) at 1:1000, mouse anti-human MCM7 [57] at 1:500, rabbit anti-human MCM10 (12251-1-AP, Protein Tech Group) at 1:1000, rabbit anti-human RECQ4 [58] at 1:1000, rabbit anti-human TOPBP1 (A300-111A, Bethyl Laboratories) at 1:1000, rabbit anti-human TRESLIN (A303-472A, Bethyl Laboratories) at 1:1000.

### Cell culture and microscopy

Human osteosarcoma cells (U2OS) were cultured in Dulbeco's Modified Eagle Medium (DMEM) Glutamax (Life Technologies) with 10% fetal bovine serum (Life Technologies) and 1% penicillin/streptomycin (Life Technologies) at 37°C in humidified 5% CO_2_ atmosphere. Chicken DT40 B-lymphoma cells were cultured in Roswell Park Memorial Institute-1640 Medium (RPMI-1640) Glutamax (Life Technologies) with 10% fetal bovine serum (Life Technologies), 1% chicken serum (Life Technologies) and 1% penicillin/streptomycin (Life Technologies) at 39.5°C in humidified 5% CO_2_ atmosphere with doxycycline (1 μg/ml, Sigma) where indicated. For nocodazole arrest, cells were pre-treated with doxycycyline for 80 h and then nocadazole was added to the final concentration of 500 ng/ml for 16 h. Microscopy was performed with a Nikon Eclipse 80i, using x100 (NA 1.40) or x60 (NA 1.40) objectives and Nikon ACT-1 version 2.62 software (Nikon).

### Cell transfection

For transient transfections of U2OS cells, 70-80% confluent cultures in 10 cm dishes were transfected using 30 μl of Lipofectamine 2000 reagent (Life Technologies), 1 ml OPTI-MEM medium (Life Technologies) and 6 μg of DNA. Cells were analyzed or harvested 48 h post-transfection. For siRNA treatments, 10^5^ U2OS cells were transfected by mixing with siRNA solution (10 nM siRNA (Sigma), 5 μl Lipofectamine 2000 (Life Technologies) and 400 μl OPTI-MEM media (Life Technologies)). After 72 h, cells were transfected with the *RECQ4* cDNA. The *RECQ4* siRNAs targeted the 3′UTR; RECQ4-1:5′-GGAACGAGGAGGCUCCAAAdTdT-3′ and 5′-UUUGGAGCCUCCUCGUUCCdTdT-3′; RECQ4-2: 5′-GUUGUCAGAGGCUAGGGCAdTdT-3′ and 5′-UGCCCUAGCCUCUGACAACdTdT-3′. To obtain stably expressing chicken clones, 10^7^ DT40 cells were harvested and washed once with phosphate buffered saline (PBS), re-suspended in 500 μl of PBS. Approximately 25 μg of linearized plasmid DNA was added to the cells, and the mixture was incubated for 10 min on ice. Cells were then transfected in 0.4 cm cuvettes (Bio-Rad) in Gene Pulser XCell with CE Module (Bio-Rad) at 300 V and 600 μF settings, and were then incubated on ice for an additional 10 min. Transfected cells were then transferred to 20 ml pre-warmed medium. 16-18 h post-transfection, cells were exposed to geneticin (Life Technologies) at 2 mg/ml and transferred to 96 well plates to obtain single colonies.

### Protein purification

A full length RECQ4 construct was a kind gift from Dr Yilun Liu. RECQ4 was purified essentially by the procedure described by [34]. The protein was expressed in Rosetta pLysS cells following growth at 18°C in medium containing 0.1 mM IPTG for 16h. The cells were harvested and lysed in buffer A (50 mM potassium phosphate, pH 8, 300 mM KCl, 10% glycerol, 0.5 mM TCEP, 0.5% Triton X-100, 1 tablet/50 ml Complete Inhibitor cocktail EDTA Free (Roche), 50 U/ml Benzonase, 1 mM PMSF, 0.1 mg/ml lysozyme). The cell suspension was lysed using a French press and clarified by centrifugation. The cleared lysate was loaded onto HQ sepharose beads (GE Healthcare) and the flow through was collected and 0.1% (W/V) polyethyleneimine (PEI) was added and stirred for 30 min at 4°C. The precipitate was clarified by centrifugation and the supernatant was loaded on to a HiTrap HP column (GE Healthcare). The column was washed with buffer B (50 mM potassium phosphate, pH 6.0, 500 mM KCl, 10 % glycerol, 0.5 % Triton X-100, 0.5 mM TCEP, 10 mM imidazole), and the bound protein was eluted with Buffer B containing 1 M imidazole. The eluted RECQ4 protein was buffer exchanged with 50 mM Tris pH 7.5, 500 mM KCl, 10% glycerol and 0.5 mM TCEP (Buffer C) and was then incubated with 100-120 μl FLAG M2 beads (Sigma) overnight at 4°C. The bound protein was washed with Buffer C and eluted with Buffer C containing 200-300 μg/ml FLAG peptide and buffer exchanged with Buffer C.

Fragments of MBP-RECQ4-HIS 1-400 and 1-400 Δ65-145 were purified from *E. coli* BL21(DE3) pLysS cells previously induced with 0.1 mM IPTG at OD­_600_ = 0.6 for 16-20 h at 16°C. For MBP-RECQ4-HIS fragments, the cell pellet was re-suspended in lysis buffer (50 mM Tris-HCl, pH 7.5, 10% sucrose, 2 mM EDTA, 200 mM KCl, 0.01% NP-40, 1 mM 2-mercaptoethanol) supplemented with protease inhibitors (aprotinin, chymostatin, leupeptin, pepstatin A and benzamidine hydrochloride at 5 mg/ml each). Cells were then lysed by sonication, and the supernatant was clarified by centrifugation at 100,000g for 1 h at 4°C. MBP-RECQ4-HIS was precipitated using HIS-Select nickel affinity gel (Sigma) at 4°C overnight. Beads were washed with buffer K (20 mM K_2_HPO_4,_ 10% glycerol, 0.5 mM EDTA, 150 mM KCl, 0.01% NP-40, 1 mM β-mercaptoethanol) containing 10 mM imidazole. The bound protein was eluted by buffer K containing increasing concentrations of imidazole (150–1000 mM). Fractions containing MBP-RECQ4-HIS were incubated with amylose beads (NEB) at 4°C for 1 h. The beads were then washed with buffer K and proteins eluted with buffer K supplemented with 10 mM maltose. Pooled fractions containing MBP-RECQ4-HIS protein were loaded onto MonoS column (GE Healthcare) and eluted using salt gradient (150-1000 mM) in buffer K. Pure fractions of MBP-RECQ4-HIS were concentrated using VivaSpin-2 (GE Healthcare).

For GST proteins (GST and GST-MCM10) *E. Coli* cells were re-suspended in GST buffer (20 mM HEPES pH 7.5, 200 mM KCl, 10% glycerol, 2 mM β-mercaptoethanol, 1 mM EDTA) supplemented with protease inhibitors (Roche) and lysed by addition of sodium lauroyl sarcosinate to final concentration of 0.8% for 10 min at 4°C on a rotating wheel. The lysate was then gently sonicated to shear genomic DNA and Triton X-100 was added to final concentration of 0.9 % to reduce protein-protein interactions. The lysate was rotated for 5 min at 4°C, before insoluble material was pelleted at 21 000 g for 15 min at 4°C. The cleared lysate was then diluted with GST buffer in 1:1 ratio to reduce detergent concentration and mixed with GSH beads. Binding of GST-proteins was performed for 30 min at 4°C on a rotating wheel. Resin was then washed extensively with GST buffer containing 0.5% Triton X-100. For GST pull-downs appropriate amount of beads containing GST or GST-MCM10 (equal microgram amounts of the GST protein) was taken for the assay. For peptide array GST-MCM10 was eluted with GST buffer (pH 8.5) containing 30 mM reduced glutathione.

### Surface plasmon resonance

Surface Plasmon resonance was performed using a BIAcore T100 (GE Healthcare) instrument at 25°C. GST MCM10 (30-50 μg/ml) was immobilized to a level of 500 response units using amine-coupling chemistry onto a CM3 chip. Two different flow rates (30 and 60 μl/min) were used to study the interaction with the analyte (RECQ4) interaction. HBS buffer (10 mM HEPES, pH 7.4, 150 mM NaCl, 0.5 mM TCEP, 0.05% Tween20) was used for dissociation. The sensograms were globally analyzed with the BIACORE T200 evaluation software (Biacore AB).

### Pull-down assays

For pull-down of YFP-tagged proteins, U2OS cells (8×10^6^) expressing YFP-RECQ4 variants were lysed in pull-down buffer (10 mM Tris pH 7.5, 200 mM KCl, 10% glycerol, 2 mM MgCl_2_, 0.5mM DTT, 0.5% NP-40) supplemented with protease, phosphatase inhibitors (Roche) and benzonase (250 U/ml; Sigma) for 1 h at 4°C on a rotating wheel. Lysates were then gently sonicated to shear cell debris and insoluble material was spun at 21,000 g for 15 min at 4°C. Protein concentration was then determined using BCA assay (Thermo Scientific) and approximately 1-2 mg of total cell extract was mixed with previously prepared GFP-Trap beads (Chromotek). Briefly, 30 μl of GFP-Trap beads were washed with pull-down buffer and blocked for 1 h with 3 mg/ml of BSA in the same buffer. Lysate-beads mixture was then incubated for 2 h at 4°C on a rotating wheel. Resin was then extensively washed with pull-down buffer, protein complexes eluted in Laemmli buffer for 10 min at 95°C and analyzed by immunoblotting.

For GST pull-downs, beads with bound GST and GST-MCM10 proteins (approximately 1 μg of GST proteins) were incubated with purified recombinant HIS-RECQ4 (approximately 1 μg) or fragments of MBP-RECQ4-HIS in YFP pull-down buffer for 1 h at 4°C on a rotating wheel. For pull-down of MCM10 interaction domains of RECQ4 as HIS-fusions, GST or GST-MCM10 containing beads were incubated with bacterial cell lysates expressing RECQ4 domains previously prepared as described in section Protein Purification. Beads were then extensively washed with YFP pull-down buffer, re-suspended in Leammli buffer. Bound proteins were eluted by boiling for 10 min at 95°C and analyzed by immunoblotting.

### Peptide array analysis

A peptide array of RECQ4 1-340 with 10 amino acids per peptide and 5 or 9 amino acids overlap was purchased from Kinexus (Vancouver, Canada). The peptides were spotted onto a membrane, which was incubated in 100% methanol for 10 min at room temperature and then washed three times with Tris Buffered Saline (TBS) for 5 min at room temperature. The membrane was then incubated in blocking buffer (4% skimmed milk, 5% sucrose in TBS containing 0.05% Tween 20 (TBS-T)) for 2 h at room temperature. The membrane was washed once in TBS-T for 5 min at room temperature and then incubated with purified GST-MCM10 at 0.1 μg/ml in blocking buffer for 1 h at 4°C, followed by three 5 min washes in TBS-T and O/N incubation with MCM10 antibodies at 4°C. After three 5 min washes in TBS-T, the membrane was incubated with secondary antibodies, washed three times for 5 min at room temperature and the signal was developed using an ECL kit (Thermo Scientific) and X-ray film (GE Healthcare).

### Cell fractionation

Approximately 10^6^ of U2OS transiently transfected cells were lysed in 70 μl of pull-down buffer supplemented with protease and phosphatase inhibitors (Roche) for 10 min on ice. The cell lysate (30 μl) was mixed with 30 μl of pull-down buffer and 30 μl of 3x Laemmli buffer (whole cell extract). Another 30 μl of lysate was centrifuged at 15,000 g for 10 min at 4°C, and the supernatant was mixed with 30 μl of pull-down and 30 μl of 3x Laemmli buffer (soluble fraction). The residual pellet was washed once with 1 ml of pull-down buffer, centrifuged again, and the pellet was re-suspended in 30 μl of pull-down buffer. Chromatin-bound proteins were released by the addition of 30 μl of pull-down buffer supplemented with benzonase (250 U/ml), incubated for 30 min at 4°C and mixed with 30 μl of 3x Leammli buffer (insoluble fraction). All samples were then boiled for 10 min at 95°C and analyzed by immunoblotting.

### Flow cytometry

Approximately, 0.5-1×10^6^ of DT40 cells were harvested by centrifugation and re-suspended in 0.25 ml of PBS. 0.5 ml of −20°C absolute ethanol was then added drop-wise to the cells while gently vortexing. Cells were fixed for at least 30 min, the ethanol was removed, and the cells were washed twice with PBS and incubated in pre-warmed RPMI 1640 medium at 37°C for 30 min. After removal of the medium, the cell pellet was treated with RNase A at 100 μg/ml and propidium iodide at 40 μg/ml in PBS for 30 min. Flow cytometry was performed using FACS Caliber instrument (Beckman-Coulter).

### DNA fiber analysis

DT40 cells were treated with doxycyline at 1 μg/ml for 96 h prior to DNA fibre isolation. All steps were taken to protect degradation of IdU (Sigma) and CldU (Sigma) by sunlight. Approximately, 7.5 × 10^5^ cells in 1 ml of medium were labelled with 25 mM IdU for 20 min at 37°C followed by labelling with 250 mM CldU for 20 min at 37°C. After labelling, cells were harvested by centrifugation at 1200 rpm for 5 min, and then re-suspended in ice cold PBS buffer. The cells (2 μl) were then spotted onto glass slide (Superfrost, Thermo Fisher Scientific) and after ~5-7 min cells were mixed with 7 μl of lysis buffer (200 mM Tris-HCl, pH7.4, 50 mM EDTA, 0.5% SDS). Cells were left to lyse for additional 2 min and slides were then tilted at an angle of 15°. After the drop reached the bottom of the slide, preparations were air-dried and fixed with methanol:acetic acid (3:1) solution for 10 min and air-dried. Slides were then washed with water and the DNA was denatured in 2.5 M HCl for 1.5 h, followed by 3 x water and 3 x PBS washes. Preparations were then incubated with anti-IdU and anti-CldU antibodies in total volume of 30 μl in 5% BSA for 1 h at room temperature. After 3 x PBS wash, slides were incubated with secondary antibodies in 5% BSA for 1 h at room temperature, washed 3 x PBS, mounted with Vectashield (Vectorlabs) and analyzed by microscopy.
